# Understanding the impact of structural modifications at the NNAT gene’s post-translational acetylation site: in silico approach for predicting its drug-interaction role in anorexia nervosa

**DOI:** 10.1007/s40519-023-01618-4

**Published:** 2023-11-21

**Authors:** Muhammad Bilal Azmi, Areesha Jawed, Syed Danish Haseen Ahmed, Unaiza Naeem, Nazia Feroz, Arisha Saleem, Kainat Sardar, Shamim Akhtar Qureshi, M. Kamran Azim

**Affiliations:** 1 Department of Biochemistry, Dow Medical College, Dow University of Health Sciences, Karachi, Pakistan; 2 Dow Medical College, Dow University of Health Sciences, Karachi, Pakistan; 3 Department of Biochemistry, University of Karachi, Karachi, Pakistan; 4 Department of Chemistry, Bahria College NORE-1, Karachi, Pakistan; 5 Department of Biosciences, Mohammad Ali Jinnah University, Karachi, Pakistan

**Keywords:** Anorexia nervosa, Acetylation, In silico, Neuronatin, Post-translational modification

## Abstract

**Purpose:**

Anorexia nervosa (AN) is a neuropsychological public health concern with a socially disabling routine and affects a person’s healthy relationship with food. The role of the NNAT (Neuronatin) gene in AN is well established. The impact of mutation at the protein’s post-translational modification (PTM) site has been exclusively associated with the worsening of the protein’s biochemical dynamics.

**Methods:**

To understand the relationship between genotype and phenotype, it is essential to investigate the appropriate molecular stability of protein required for proper biological functioning. In this regard, we investigated the PTM-acetylation site of the NNAT gene in terms of 19 other specific amino acid probabilities in place of wild type (WT) through various in silico algorithms. Based on the highest pathogenic impact computed through the consensus classifier tool, we generated 3 residue-specific (K59D, P, W) structurally modified 3D models of NNAT. These models were further tested through the AutoDock Vina tool to compute the molecular drug binding affinities and inhibition constant (*Ki*) of structural variants and WT 3D models.

**Results:**

With trained in silico machine learning algorithms and consensus classifier; the three structural modifications (K59D, P, W), which were also the most deleterious substitution at the acetylation site of the NNAT gene, showed the highest structural destabilization and decreased molecular flexibility. The validation and quality assessment of the 3D model of these structural modifications and WT were performed. They were further docked with drugs used to manage AN, it was found that the ΔGbind (kcal/mol) values and the inhibition constants (*Ki*) were relatively lower in structurally modified models as compared to WT.

**Conclusion:**

We concluded that any future structural variation(s) at the PTM-acetylation site of the NNAT gene due to possible mutational consequences, will serve as a basis to explore its relationship with the propensity of developing AN.

**Level of evidence:**

No level of evidence—open access bioinformatics research.

## Introduction

Eating disorders (EDs) are characterized as a neuropsychological public health concern with socially disabling routines and cause an unhealthy relationship with food [1]. Anorexia nervosa (AN), a type of ED, causes neuropsychiatric relational impedance for lack of appetite [2]**.** Other manifestations of the disease include pronounced weight loss with lower health-related quality of life (HRQoL) [3]. Studies from Western countries suggest a prevalence of approximately 0.2–0.8% and an incidence of 4–8 new cases per year per 100,000 individuals [4]. The condition is 10 to 20 times more likely to occur in women than in men, and the average age of disease onset is between 14 and 18 years [4]. According to the most recent comprehensive meta-analysis from Japan, the mortality rate of AN over 10 years was found to be around 5–6%, considerably higher than any other psychiatric disorder, while the 5-year recovery rate has been reported to be 66.8% [5].

By far, significant progress has not been observed in establishing the molecular basis of AN in humans, to identify any gene-level mutation or loss at post-translational modifications site (PTMs) that confers disease risk, remaining elusive [6, 7]. PTMs are protein chemical modifications that regulate activity, localization, and interaction with other cellular molecules such as proteins, nucleic acids, lipids, and cofactors [8]. Besides this, PTMs like acetylation, glycosylation, hydroxylation, phosphorylation, etc., play an imperative role in the function of protein homeostasis proteins and are perilous in the development and progress of disease [6–8]. PTMs are involved in the diversification of the proteome, as there are ~ 20–25,000 protein-encoding genes, while the proteome is estimated to be over 1,000,000 proteins in size [6, 9].

PTMs in proteins not only influence secondary structural level changes, but also have a significant impact on the protein's tertiary level, which in turn influences the protein's biochemical and physiological behavior [8, 9]. In addition, these secondary-level modifications are linked to variations in the protein’s homeostasis and have wide-ranging effects on their ability to interact with other proteins [7, 9]. Owing to the impact of PTMs on gene expression, it was earlier reported that these modifications were linked to altering the structure of chromatin as well as recruiting histone modifiers [10]. PTMs in the gene(s) mediate various biological functions such as processes involved in DNA damage and repair, transcriptional regulation, chromosome packaging, and others [8, 9].

Germline genetic variations located in the coding region (missense rare-coding single nucleotide polymorphisms ‘SNPs’) of genes have been significantly associated with complex traits or pathogenicity, like type 2 diabetes, cancer, cardiovascular diseases, etc. [11]. It is well postulated that these missense SNPs, particularly at PTMs-sites, may modify amino acid residues, thereby collectively and significantly influencing some serious biochemical or physiological processes by causing protein structural disorders [8, 11]. They can potentially disrupt protein macromolecular interactions [7, 8] or affect the stability of protein [11]. It is a challenge for the scientific community to logically analyze such missense variations, especially at the PTM acetylation site, to discern how they structurally and functionally affect the protein. Unfortunately, there is still a dearth of effective techniques for functional annotation and prediction of these coding variants, particularly for changes that could potentially relate to PTMs.

The gene(s) level association and etiology of AN remain uncertain while several kinds of research has been conducted to probe this condition genetically. Recently, the Neuronatin (NNAT) gene was reported to be associated with health and disease conditions [12]. For instance, Lombardi et al*.* reported that any variation in gene expression status or any dysregulation in the (canonical isoform) brain-specific NNAT leads to the development of AN [13]. Extending this deduction, Sanger sequencing of the promoter region of NNAT by Ceccarrini et al. in 2021 further confirmed the contribution of this gene in the development of AN [14]. In 2022, Azmi et al. identified rs539681368 (C30Y), a high-risk deleterious non-synonymous SNP, and the possibility of its PTM site for acetylation was also recognized, i.e., the K59 residue, having significant drug-binding energies and the commonest interaction observed [15].

In this study, we investigate the impact of structural modifications at NNAT’s PTM-acetylation site (K59) located in the cytoplasmic domain. We also predict the impact of other amino acids substituted instead of LYS at the PTM-acetylation site (59th) position on the functional stability of the protein as well as the computation of pathogenicity scores. Individually, the wild-type (WT) and the most pathogenic 3D-models obtained through residue-specific substitution (of NNAT gene) having the most pathogenic scores with prominent structural modifications at the PTM-acetylation site were investigated in terms of in silico molecular drug-binding affinities and estimation of inhibition (*K*_*i*_) constant with the therapeutic compounds of AN. Using the molecular drug binding energies that we had initially obtained, the significant role of PTM site and their associated structural modifications were considered to predict the impact of structural modifications on drug interaction at the NNAT gene’s PTM acetylated site.

## Methodology

**Fig. 1:**
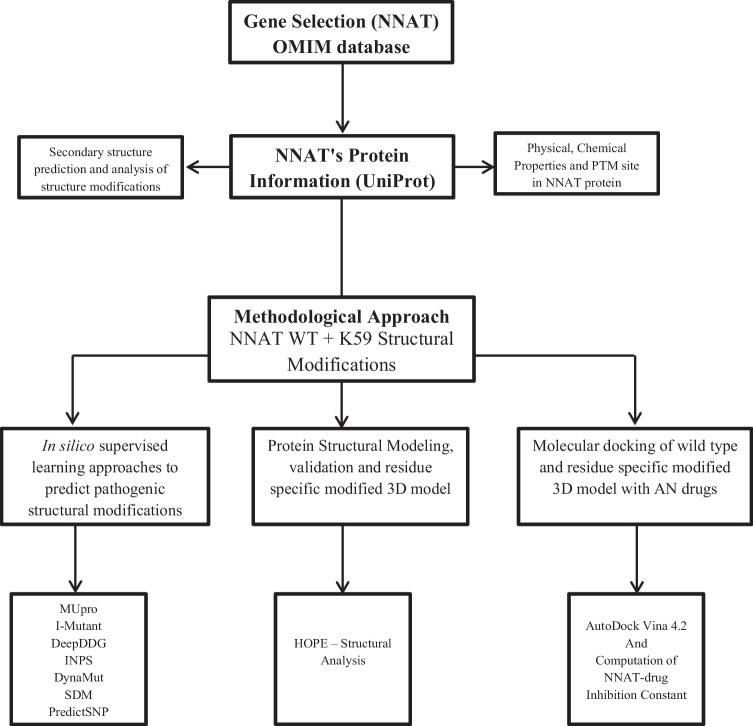
Flowchart representing study’s work scheme

In Fig. 1, the overall work-scheme of the present study is represented.

### Extraction of gene and protein-level information

The publically available genomic data on the human NNAT gene were collected from the Gene database of the NCBI (National Center for Biological Information) website (https://www.ncbi.nlm.nih.gov/gene). Information regarding cytogenic location, HGNC-approved gene symbol, GenBank accession detail, gene identifier, and other information was extracted from OMIM (Online Mendelian Inheritance in Man; https://www.omim.org/) database repository for comprehensive information on human genes and genetic phenotypes with their relationships. The protein-level information was accessed through the protein accession number and UniProtKB database (https://www.uniprot.org/help/uniprotkb).

### Sequence-based computation of physical and chemical properties and PTM acetylation site identification

The sequence-based computation of several chemical and physical parameters for the NNAT protein was done through *Expasy* ProtParam tool (https://web.expasy.org/protparam/). These parameters included the amino acid composition, molecular weight, atomic composition, theoretical *pI*, extinction coefficient, estimated half-life, instability index, aliphatic index, and grand average of hydropathicity (GRAVY). The prediction of the PTM acetylation site was investigated with the aid of deep learning *MusiteDeep* web server (https://www.musite.net) [16].

### Protein secondary structure prediction, computation of torsional angles, and analysis of structural modifications associated at the PTM acetylated site

To investigate the impact of the substitution of amino acid at the PTM-acetylation site (59^th^ position) of the NNAT gene based on protein secondary structure, surface accessibility, percent disordered residue, torsional angles, i.e., phi (*ϕ*) psi (*ψ*) were computed from NetSurfP-2.0 web server (https://services.healthtech.dtu.dk/service.php?NetSurfP-2.0) of Technical University of Denmark (DTU) [17]. To investigate the possible 3D structural variation associated with the PTM-acetylation site, the Cologne University Protein Stability Analysis Tool (CUPSAT) web server (http://cupsat.tu-bs.de/) was applied. This tool obtained transformation in the specific atom(s) potential and torsion angle potential in terms of ΔΔG (kcal/mol) [18]. This sequence-based strategy resulted in the computation of the free energy difference of unfolding conformational state between wild type (WT)/ native protein with the rest of 19 modified protein 3D structural models. For the analysis of different structural modifications at the PTM-acetylation site, HOPE (Have (y) Our Protein Explained: https://www3.cmbi.umcn.nl/hope/) web-server was used [19]. This tool predicted the effects of the modifications linked with a mutation on the protein structure and its accompanying function. It gathered and combined information from several web services and databases to create a report which included findings, statistics, and animations. Protein sequence and particular residue specific variations were the input criteria for the HOPE algorithm. [19].

### In silico supervised learning approaches to investigate the hypothetical pathogenic structural prediction and its impact on protein function dynamics

To predict the free energy difference causing overall structural destabilization at the PTM-acetylation site, different machine learning strategies were computed. These were originally used to investigate the impact of mutations on overall protein stability and its 3D function. The first approach was the MUpro machine learning program, used through SVM (Support Vector Machines) and NN (Neural Networks) [20]. The second approach was the I-Mutant (version 3) predictor, which computed the automatic prediction of protein stability changes upon single substitution at pH 7 and 25 °C temperature [21]. The third approach was INPS-MD (Impact of Non-synonymous mutations on Protein Stability Multi Dimension), https://inpsmd.biocomp.unbito.it [22]. Another approach was based on the use of the DeepDDG web server which predicted the stability change of protein residue-specific substitution using neural networks [23]. SDM (Site Directed Mutator) was another computational method for analyzing the variation of amino acid replacements occurring at particular structural environments that were permitted within the family of homologous proteins with known 3-D structures, turning them into substitution probability tables [24]. To analyze and visualize protein dynamics by sampling conformations, and to evaluate the effects of residue modifications / possible mutations on protein dynamics and stability brought by changes in vibrational entropy, DynaMut, a web server that implemented two independent, well-established normal mode techniques, was employed [25].

To further investigate and validate the expected accuracy of possibly deleterious structural modifications due to the substitution of different amino acids at the PTM-acetylation site, the PredictSNP1.0 consensus classifier predictor approach was used (https://loschmidt.chemi.muni.cz/predictsnp1/). This web-server tool was used to investigate the effects of SNPs/structural substitution leading to any significant change in protein function.

### Structural modeling and validation of wild-type and PTM-acetylation site-based structurally modified 3D pathogenic models

As no 3D structural information/coverage was reported at Protein Data Bank (PDB) [26] level, an ab initio 3D protein structural prediction strategy was selected to build a 3D model from Zhang Lab’s [27] web server. For selecting the optimal 3D model, validation of all five generated models was performed through PROCHECK stereo-chemical assessment. Additionally, structural analysis based on the quality factor and Ramachandran plot was performed [28]. The probabilities for the occurrence of structural error and z-score values estimation were performed through Structure Validation Server (SAVES) [28].

After the identification of the PTM acetylation (LYS) residue/site at the 59th position, the remaining 19 3D models having a residue-specific substitution at the 59th position were constructed through the UCSF Chimera visualization program [29].

### Molecular docking of WT and structurally modified (pathogenically predicted) 3D models with therapeutic drugs of AN

After a thorough literature search from the DrugBank database, the 2D structures of the drugs used to manage AN were downloaded from the PubChem database. The obtained 2D compound format was converted into a 3D PDB format through the Open Babel tool [30]. The process of energy minimization of each 3D drug was performed by the FROG2 tool [31]. The potential drug–protein binding cavities were detected through the CB-Dock (Cavity binding Docking) approach and COACH server, for the identification of key active interaction residues in protein [32]. The 3D wild-type model of NNAT protein with 3D compounds of AN drugs were investigated based on binding affinities. The Consensus Interacting Residues (CIR) were inspected with the aid of a 2D plot generated through Discovery studio and were selected based on their mutual appearance in cavities of the highest energetic grades [33]. Residue-specific virtual screening and molecular docking of WT and structurally modified NNAT proteins with all 3D AN drugs (ligands) were performed through AutoDock Vina 4.2 software, [34] which predicted pathogenic effect models (with structural modification at PTM residue substitution). This experimental procedure was performed to investigate the inhibitory impact and the binding pattern potentially residing at the PTM acetylation (K59) site with AN drugs related to WT-conserved protein and structurally modified (predicted pathogenic effect) 3D models. To find out the association of binding energies (ΔG) with the inhibition constant (*K*_*i*_) of WT and the structurally modified model we used a linear regression model in which we set *K*_*i*_ values as the dependent variable because of its ΔG dependent computation, and ΔG was the independent variable through online web-server of https://www.graphpad.com/quickcalcs/ and https://www.statskingdom.com/index.html.

## Results

### Compositional breakdown and residue-based probability estimation

The estimated molecular weight of NNAT was 9236.83 Dalton with a theoretical *pI* of 10.17. The highest (12.3%, 11.1%) composition of alanine and arginine was observed while lysine was 1.2%, *i.e.,* only one LYS at 59th position. The total number of negatively charged (D + E) residues were 4 and positively charged residues were (R + K) 10. The atomic composition of NNAT protein showed carbon (C = 424), hydrogen (H = 662), nitrogen (N = 118), oxygen (O = 108), and sulphur (S = 3) with a total number of 1315 atoms. The extinction coefficient was 17,085 M^−1^ cm^−1^, at 280 nm measured in H_2_O, when absorbance 0.1% (= 1 g/l) 1.850, assuming all pairs of cysteine residues form cysteine. The extinction coefficient was 16,960 M^−1^ cm^−1^, when absorbance 0.1% (= 1 g/l) 1.836, assuming all pairs of cysteine residues are reduced.

The N-terminal of the sequence is methionine (M) and the estimated half-life is 30 h (mammalian reticulocyte, in vitro), > 20 h (yeast, in vivo), > 10 h (*Escherichia coli*, in vitro) with an aliphatic index of 100. The grand average of the hydropathicity index (GRAVY) is 0.217. The predicted trans-membrane helix (TMH) was 1, which started with the 13th ILE and ends at the 35th GLY, which showed the expected number of residues in TMH was 22.4976.

### Secondary structural prediction and computation of torsional angles

To investigate the secondary structure prediction of the NNAT protein; the impact of residue substitution at the PTM-acetylation site on secondary structure composition, torsional angles, and energetic potentials on overall protein structure was computed. Any substitution in terms of CYS, PHE, TRP, and TYR showed overall protein stability, while the residue substitution at the LYS59th position was contrary to this function (see Table 1).

**Table1:** Impact of structural modifications through residue-specific substitution (RSS) at PTM (K59) site on secondary structure, torsional angles and energetic potentials of protein

RSS	RSA (%)	ASA (Å)	SS_3_	SS_8_	Phi ( ϕ)	Psi ( ψ)	Intrinsically disordered regions (%)	Torsion angle potentials	Overall stability	Predicted ΔΔG (kcal/mol)
Alanine	24	31	Helix	α-helix	− 65	− 39	1	Favorable	Destabilizing	− 1.3
Arginine	37	102	Helix	α-helix	− 67	− 38	1	Favorable	Destabilizing	− 0.15
Asparagine	39	76	Helix	α-helix	− 69	− 30	1	Unfavorable	Destabilizing	− 1.23
Aspartic acid	34	66	Helix	α-helix	− 68	− 35	1	Unfavorable	Destabilizing	− 0.96
Cysteine	16	26	Helix	α-helix	− 68	− 38	1	Unfavorable	Stabilizing	0.05
Glutamic acid	39	86	Helix	α-helix	− 68	− 38	1	Favorable	Destabilizing	− 0.56
Glutamine	37	83	Helix	α-helix	− 68	− 37	1	Favorable	Destabilizing	− 0.57
Glycine	29	30	Helix	α-helix	− 65	− 38	1	Unfavorable	Destabilizing	− 1.69
Histidine	38	85	Helix	α-helix	− 69	− 35	1	Unfavorable	Destabilizing	− 0.24
Leucine	44	81	Helix	α-helix	− 65	− 43	0	Favorable	Destabilizing	− 0.48
Isoleucine	19	38	Helix	α-helix	− 66	− 41	1	Unfavorable	Destabilizing	− 0.97
Methionine	21	46	Helix	α-helix	− 66	− 38	1	Favorable	Destabilizing	− 1.47
Phenylalanine	22	53	Helix	α-helix	− 67	− 39	1	Favorable	Stabilizing	0.03
Proline	45	64	Helix	α-helix	− 65	− 36	0	Unfavorable	Destabilizing	− 0.99
Serine	31	48	Helix	α-helix	− 70	− 37	1	Unfavorable	Destabilizing	− 1.09
Threonine	31	53	Helix	α-helix	− 70	− 40	1	Unfavorable	Destabilizing	− 0.78
Tryptophan	24	69	Helix	α-helix	− 66	− 40	1	Favorable	Stabilizing	0.37
Tyrosine	30	78	Helix	α-helix	− 68	− 41	1	Favorable	Stabilizing	0.26
Valine	20	36	Helix	α-helix	− 66	− 41	1	Unfavorable	Destabilizing	− 1.07

### Impact of structural modification through residue substitution

From HOPE inference, sequentially all 19 amino acids were analyzed to investigate the effect of residue substitution on the physical and chemical properties, hydrophobicity, spatial confirmation as well as the overall impact on the structure, and function of the protein (see Table 2). Based on HOPE, the modified residue ARG, TRP, and TYR were bigger than the WT (K59), whereas the modification with ALA, ASN, CYS, GLU, GLN, GLY, ILE, LEU, MET, THR, PRO, SER, VAL were smaller. In addition, the modification with CYS, GLY, ILE, LEU, MET, PHE, PRO, SER, THR, TRP, TYR, and VAL were found to increase the hydrophobicity. In general, this can be implicated by the differences in the hydrophobicity and size between WT and modified residue which could disrupt the H-bonding interactions with the neighboring residues; hence the protein interaction in the framework would be lost. Moreover, for the modified residue PRO, this substitution might disrupt a special conformation of protein as well as break the helical bonding at the PTM acetylation site (see Table 2).

**Table 2:** Structural Inference from HOPE server investigated on each residue specific substitution (RSS) at PTM (K59) site of NNAT protein

Structural modification at 59 position	Molecular interactions	Structural inference	PTM site analysis
Lysine		Wild type	Acetylation site at 59th position was predicted. 29–30 cysteines are Palmitoylation site. 70th (glutamine) position indicates Pyrrolidone carboxylic acid site
Alanine (A)		The modified residue is smaller than the wild-type residue. The wild-type residue charge was POSITIVE; the modified residue charge is NEUTRAL. The modified residue is more hydrophobic than the wild-type residue and located near a highly conserved position. This structural modification can result in loss of hydrogen bonds and/or disturb correct folding, hence might lead to loss of interactions	Acetylation site at 59th position was lost. 29–30 cysteines are Palmitoylation site. 54th position indicates methylation site. 70th (glutamine) position indicates Pyrrolidone carboxylic acid
Arginine (R)		The modified residue is bigger, this might lead to bumps. Any future mutation with arginine converts the wild-type residue in a residue that does not prefer α-helices as secondary structure	Acetylation site at 59th position was lost. 29–30 cysteines are Palmitoylation site. 70th (glutamine) position indicates Pyrrolidone carboxylic acid site
Asparagine (N)		The wild-type and modified amino acids differ in size. The modified residue is smaller; this might lead to loss of interactions. The modified residues (N) convert the wild-type residue in a residue that does not prefer α-helices as secondary structure	Acetylation site at 59th position was lost. 29–30 cysteines are Palmitoylation site. 54th position indicates methylation site. 70th (glutamine) position indicates Pyrrolidone carboxylic acid site
Aspartic acid (D)		The wild-type residue charge was POSITIVE, the modified residue charge is NEGATIVE, and this can cause repulsion with other residues in the protein or ligands. This modification converts the wild-type residue in a residue that does not prefer α-helices as secondary structure, lead to loss of interactions	Acetylation site at 59th position was lost. 29–30 cysteines are Palmitoylation site. 70th (glutamine) position indicates Pyrrolidone carboxylic acid site
Cysteine (C)		The modified residue is smaller than the wild-type residue. The wild-type residue charge was POSITIVE, the modified residue charge is NEUTRAL. The modified residue is more hydrophobic than the wild-type residue and does not prefer α-helices as secondary structure	Acetylation site at 59th position was lost. 29–30 cysteines are Palmitoylation site. 70th (glutamine) position indicates Pyrrolidone carboxylic acid site
Glutamic Acid (E)		The modified residue is smaller than the wild-type residue. The wild-type residue charge was POSITIVE. The modified residue charge is NEGATIVE, can cause repulsion with other residues in the protein or ligands, and might lead to loss of interactions	Acetylation site at 59th position was lost. 29–30 cysteines are Palmitoylation site. 70th (glutamine) position indicates Pyrrolidone carboxylic acid site
Glutamine (Q)		The modified residue is smaller than the wild-type residue. The wild-type residue charge was POSITIVE, the modified residue charge is NEUTRAL that does not prefer α-helices as secondary structure. This modification can cause loss of interactions with other molecules or residues	Acetylation site at 59th position was lost. 29–30 cysteines are Palmitoylation site. 54th position indicates methylation site. 70th (glutamine) position indicates Pyrrolidone carboxylic acid site
Glycine (G)		The modified residue is smaller than the wild-type residue. The wild-type residue charge was POSITIVE, the modified residue charge is NEUTRAL. The modified residue is more hydrophobic than the wild-type residue can cause loss of interactions with other molecules or residues. Glycines are very flexible and can disturb the required rigidity of the protein at this position	Acetylation site at 59th position was lost. 29–30 cysteines are Palmitoylation site. 70th (glutamine) position indicates Pyrrolidone carboxylic acid site
Histidine (H)		The wild-type residue charge was POSITIVE, the modified residue charge is NEUTRAL. The charge of the wild-type residue will be lost; this can cause loss of interactions with other molecules or residues	Acetylation site at 59th position was lost. 29–30 cysteines are Palmitoylation site. 70th (glutamine) position indicates Pyrrolidone carboxylic acid site
Isoleucine (I)		The modified residue is smaller than the wild-type residue. The wild-type residue charge was POSITIVE, the modified residue charge is NEUTRAL. The modified residue is more hydrophobic than the wild-type residue; this can result in loss of hydrogen bonds and/or disturb correct folding	Acetylation site at 59th position was lost. 29–30 cysteines are Palmitoylation site. 70th (glutamine) position indicates Pyrrolidone carboxylic acid site
Leucine (L)		The modified residue is smaller than the wild-type residue. The wild-type residue charge was POSITIVE, the modified residue charge is NEUTRAL. The mutant residue is more hydrophobic than the wild-type residue	Acetylation site at 59th position was lost. 29–30 cysteines are Palmitoylation site. 70th (glutamine) position indicates Pyrrolidone carboxylic acid site
Methionine (M)		The modified residue is smaller than the wild-type residue. The wild-type residue charge was POSITIVE, the modified residue charge is NEUTRAL. The modified residue is more hydrophobic than the wild-type residue; this might lead to loss of interactions	Acetylation site at 59th position was lost. 29–30 cysteines are Palmitoylation site. 54th position indicates methylation site. 70th (glutamine) position indicates Pyrrolidone carboxylic acid site
Phenylalanine (F)		The wild-type residue charge was POSITIVE, the modified residue charge is NEUTRAL. The modified residue is more hydrophobic than the wild-type residue that does not prefer α-helices as secondary structure. The modification introduces a more hydrophobic residue at this position which can result in loss of hydrogen bonds and/or disturb correct folding	Acetylation site at 59th position was lost. 29–30 cysteines are Palmitoylation site. 70th (glutamine) position indicates Pyrrolidone carboxylic acid site
Proline (P)		The modified residue is smaller than the wild-type residue. The wild-type residue charge was POSITIVE, the modified residue charge is NEUTRAL. The modified residue is more hydrophobic than the wild-type residue which can result in loss of hydrogen bonds and/or disturb correct folding	Acetylation site at 59th position was lost. 29–30 cysteines are Palmitoylation site. 54th position indicates methylation site. 70th (glutamine) position indicates Pyrrolidone carboxylic acid site
Serine (S)		The modified residue is smaller than the wild-type residue. The wild-type residue charge was POSITIVE, the modified residue charge is NEUTRAL. The modified residue is more hydrophobic than the wild-type residue, which can result in loss of hydrogen bonds and/or disturb correct folding	Acetylation site at 59th position was lost. 29–30 cysteines are Palmitoylation site. 54th position indicates methylation site. 70th (glutamine) position indicates Pyrrolidone carboxylic acid site
Threonine (T)		The modified residue is smaller than the wild-type residue. The wild-type residue charge was POSITIVE, the modified residue charge is NEUTRAL. The modified residue is more hydrophobic than the wild-type residue which can result in loss of hydrogen bonds and/or disturb correct folding	Acetylation site at 59th position was lost. 29–30 cysteines are Palmitoylation site. 54th position indicates methylation site. 70th (glutamine) position indicates Pyrrolidone carboxylic acid site
Tryptophan (W)		The modified residue is bigger than the wild-type residue. The wild-type residue charge was POSITIVE, the modified residue charge is NEUTRAL. The modified residue is more hydrophobic than the wild-type residue, this might lead to bumps	Acetylation site at 59th position was lost. 29–30 cysteines are Palmitoylation site. 70th (glutamine) position indicates Pyrrolidone carboxylic acid site
Tyrosine (Y)		The modified residue is bigger than the wild-type residue. The wild-type residue charge was POSITIVE, the mutant residue charge is NEUTRAL. The modified residue is more hydrophobic than the wild-type residue, which can result in loss of hydrogen bonds and/or disturb correct folding	Acetylation site at 59th position was lost. 29–30 cysteines are Palmitoylation site. 70th (glutamine) position indicates Pyrrolidone carboxylic acid site
Valine (V)		The modified residue is smaller than the wild-type residue. The wild-type residue charge was POSITIVE, the modified residue charge is NEUTRAL. The modified residue is more hydrophobic than the wild-type residue, which can result in loss of hydrogen bonds and/or disturb correct folding	Acetylation site at 59th position was lost. 29–30 cysteines are Palmitoylation site. 70th (glutamine) position indicates Pyrrolidone carboxylic acid site

### In silico computation of free energy differences due to structural modifications at the PTM acetylation site

Through in silico supervised learning approaches, the computation of free energy resulting due to PTM acetylation site structural modifications on protein showed a higher decrease in stability of protein function, while very few substitutions (like LUE, PHE, ILE, MET) reported stabilities in function for I-Mutant and MUpro ∆∆G (kcal/mol) energies. However, LEU and ILE were the only two residues which were showing the stabilities of protein functions computed through Deep-DDG and INSPS tools. Through SDM approach, the energies and the other related parameters for computing the impact of protein stability upon residue-specific structural modifications showed approximately 11 residues (N, D, C, G, H, M, F, P, S, T, V instead of lysine) with a prominent reduction in structural conformation stability (see Table 3). The highest structural destabilization was observed in K59P with a score of − 1.54 kcal/mol (see Tables 3 and 4).

**Table 3:** Analyzing the impact of amino acid substitution at PTM (K59) site on overall structural environment of NNAT protein

RSS	WT RSA^a^ (%)	WT DEPTH^b^ (Å)	WT OSP^c^	MT RSA^d^ (%)	MT DEPTH^e^ (Å)	MT OSP^f^	Predicted ΔΔG
K59A	78.2	3.3	0.2	69.5	3.1	0.33	0.80
K59R	78.2	3.3	0.2	74.2	3.5	0.22	0.26
K59N	78.2	3.3	0.2	79.6	3.4	0.26	− 0.86
K59D	78.2	3.3	0.2	65.4	3.4	0.31	− 0.76
K59C	78.2	3.3	0.2	77.5	3.3	0.27	− 0.33
K59E	78.2	3.3	0.2	62.6	3.5	0.29	0.99
K59Q	78.2	3.3	0.2	79.6	3.4	0.23	0.44
K59G	78.2	3.3	0.2	93.0	3.6	0.35	− 0.77
K59H	78.2	3.3	0.2	79.6	3.5	0.22	− 0.45
K59I	78.2	3.3	0.2	71.0	3.5	0.28	0.19
K59L	78.2	3.3	0.2	77.3	3.5	0.23	0.69
K59M	78.2	3.3	0.2	74.0	3.3	0.22	− 0.05
K59F	78.2	3.3	0.2	64.2	3.5	0.26	− 0.28
K59P	78.2	3.3	0.2	46.8	3.5	0.34	− 1.54
K59S	78.2	3.3	0.2	69.6	3.2	0.31	− 0.77
K59T	78.2	3.3	0.2	66.3	3.3	0.32	− 0.64
K59W	78.2	3.3	0.2	77.0	3.6	0.18	0.03
K59Y	78.2	3.3	0.2	78.2	3.4	0.19	0.15
K59V	78.2	3.3	0.2	65.3	3.3	0.30	− 0.06

^a^ = residue relative solvent accessibility for wild type, ^b^ = residue depth for wild type, ^c^ = occluded surface packing value for wild type residue, ^d^ = relative solvent accessibility for modified residue, ^e^ = depth for modified residue, ^f^ = occluded surface packing value for modified residue

**Table 4:** Prediction of protein stability changes upon residue specific substitution (RSS) at PTM (K59) site through NMA-based analysis

RSS	DynaMut ∆∆G (kcal/mol)	NMA ENCoM ∆∆G (kcal/mol)	mCSM ∆∆G (kcal/mol)	SDM ∆∆G (kcal/mol)	DUET ∆∆G (kcal/mol)	ΔΔS Vib ENCoM (kcal.mol^−1^.K^−1^)
Alanine	0.019^S^	− 0.136^D^	− 0.537^D^	0.500^S^	− 0.134^D^	0.170^
Arginine	0.015^S^	0.044	− 0.411^D^	− 0.110^D^	− 0.091^D^	− 0.055*
Asparagine	0.441^S^	0.095^D^	− 0.183^D^	− 0.770^D^	− 0.052^D^	− 0.118*
Aspartic acid	0.336^S^	0.110^D^	0.313^S^	− 0.590^D^	0.530^S^	− 0.137*
Cysteine	− 0.246^D^	− 0.082^D^	− 0.447^D^	− 0.940^D^	− 0.500^D^	0.102^
Glutamic acid	0.329^S^	− 0.033^D^	0.411^S^	0.990^S^	0.995^S^	− 0.042*
Glutamine	− 0.012^D^	− 0.013^D^	− 0.235^D^	− 0.150^D^	0.074^S^	0.016^
Glycine	− 0.630^D^	− 0.157^D^	− 0.865^D^	− 0.830^D^	− 0.874^D^	0.197^
Histidine	− 0.059^D^	0.012^D^	− 1.264^D^	− 0.560^D^	− 1.258^D^	− 0.015*
Leucine	0.048^S^	− 0.079^D^	0.294^S^	− 0.170^D^	0.519^S^	0.099^
Isoleucine	0.221^S^	− 0.106^D^	0.294^S^	− 0.300^D^	0.484^S^	0.132^
Methionine	0.197^S^	0.103^D^	0.199^S^	− 0.350^D^	0.219^S^	− 0.129*
Phenylalanine	0.131^S^	0.051^D^	− 0.416^D^	− 0.560^D^	− 0.568^D^	− 0.064*
Proline	0.058^S^	0.061^D^	0.047^S^	− 1.290^D^	− 0.049^D^	− 0.076*
Serine	− 1.106^D^	− 0.059^D^	− 0.878^D^	− 1.420^D^	− 0.930^D^	0.074^
Threonine	0.250^S^	0.022^D^	− 0.574^D^	− 0.900^D^	− 0.470^D^	− 0.028*
Tryptophan	0.482^S^	0.306^D^	− 0.308^D^	− 0.280^D^	− 0.411^D^	− 0.382*
Tyrosine	0.432^S^	0.301^D^	0.000^S^	− 0.630^D^	− 0.185^D^	− 0.376*
Valine	0.213^S^	0.015^D^	0.047^S^	− 0.590^D^	0.154^S^	− 0.019*

^S^ = Stabilizing, ^D^ = destabilizing, ^ = increase of molecular flexibility, * = decrease of molecular flexibility, NMA = normal mode analysis, ENCoM = Elastic Network Contact Model, mCSM = mutation Cutoff Scanning Matrix, SDM = Site Directed Mutator, ΔΔS Vib ENCoM = Vibrational Entropy Energy Between Wild-Type and Mutant

To validate our findings, PredictSNP in silico approach was recruited to sort the possible deleterious or pathogenic structural modifications. We only selected those possible amino acid residues which had overall deleterious scores in all tools implemented through this web server. ASP, PRO, and TRP were the three only amino acids found with the highest expected accuracy scores for pathogenic impact (see Table 5). This demonstrates that substitution at LYS59 with any of these three amino acids may have a deleterious effect (see Table 5).

**Table 5:** In silico prediction through consensus classification of expected residue substitution or variation at PTM Actylation (K59) site of *NNAT* gene

RSS	PredictSNP (%)	SNAP2	PhD-SNP (%)	PolyPhen-1 (%)	PolyPhen-2 (%)	SIFT (%)	SNAP (%)	MAPP (%)	PROVEAN*
Alanine	61^D^	30 (66%)	51^N^	67^N^	50^D^	79^D^	72^D^	86^D^	− 4.5
Arginine	60^N^	22 (63%)	55^N^	67^N^	50^D^	79^D^	58^N^	46^D^	− 2.25
Asparagine	76^D^	49 (71%)	55^N^	59^D^	55^D^	79^D^	81^D^	66^D^	− 3.75
Aspartic acid	87^D^	65 (80%)	73^D^	59^D^	55^D^	79^D^	81^D^	78^D^	− 4.5
Cysteine	87^D^	37 (66%)	61^D^	74^D^	65^D^	79^D^	81^D^	77^D^	− 6
Glutamic acid	61^D^	56 (75%)	51^N^	67^N^	50^D^	79^D^	81^D^	81^D^	− 3
Glutamine	76^D^	35 (66%)	55^N^	59^D^	55^D^	79^D^	62^D^	66^D^	− 3
Glycine	76^D^	58 (75%)	51^N^	59^D^	55^D^	79^D^	81^D^	84^D^	− 5.25
Histidine	87^D^	36 (66%)	59^D^	74^D^	60^D^	79^D^	72^D^	72^D^	− 4.5
Isoleucine	87^D^	33 (66%)	86^D^	74^D^	60^D^	79^D^	81^D^	86^D^	− 6
Leucine	87^D^	35 (66%)	61^D^	59^D^	55^D^	79^D^	72^D^	75^D^	− 5.25
Methionine	87^D^	27 (63%)	58^D^	74^D^	65^D^	79^D^	81^D^	76^D^	− 4.5
Phenylalanine	76^D^	48 (71%)	51^N^	74^D^	60^D^	79^D^	81^D^	77^D^	− 6
Proline	87^D^	64 (80%)	61^D^	59^D^	60^D^	79^D^	89^D^	86^D^	− 4.5
Serine	72^D^	42 (71%)	61^D^	67^N^	55^D^	79^D^	72^D^	78^D^	− 3.75
Threonine	79^D^	44 (71%)	45^N^	59^D^	55^D^	79^D^	81^D^	84^D^	− 4.5
Tryptophan	87^D^	57 (75%)	68^D^	74^D^	65^D^	79^D^	85^D^	81^D^	− 6
Tyrosine	87^D^	46 (71%)	59^D^	74^D^	60^D^	79^D^	81^D^	86^D^	− 5.25
Valine	76^D^	33 (66%)	55^N^	59^D^	55^D^	79^D^	81^D^	82^D^	− 5.25

^D^Indicates the deleterious prediction of protein, ^N^indicates the neutral prediction of protein, * = all values are representing pathogenic states, In SNAP2: score ≥ 50 represents deleterious predictions

### 3D modeling and assessment of WT and pathogenically predicted PTM acetylation site structural variants

With the aid of the QUARK algorithm, five WT models were built according to rank and the best 3D model of NNAT-WT was selected based on the Ramachandran plot and quality factor analyses. After the selection and assessment of the WT model of NNAT protein, residue-specific substitution was employed through the UCSF Chimera tool for building three structural modifications at the PTM acetylation site (K59) residue. The first 3D-structural modification was the substitution of lysine (K59D) with aspartic acid followed by a proline (K59P) and tryptophan (K59W), respectively. The best 3D-models selected for K59D, K59P, and K59W structural modifications had probability scores of 0.4638 (*Chi1* = − 71.5 and *Chi2* = − 15.1), 0.9598 (*Chi1* = − 30.8 and *Chi2* = 40.1), and 0.240 (*Chi1* = 179.4 and *Chi2* = 85.8), respectively.

**Fig. 2:**
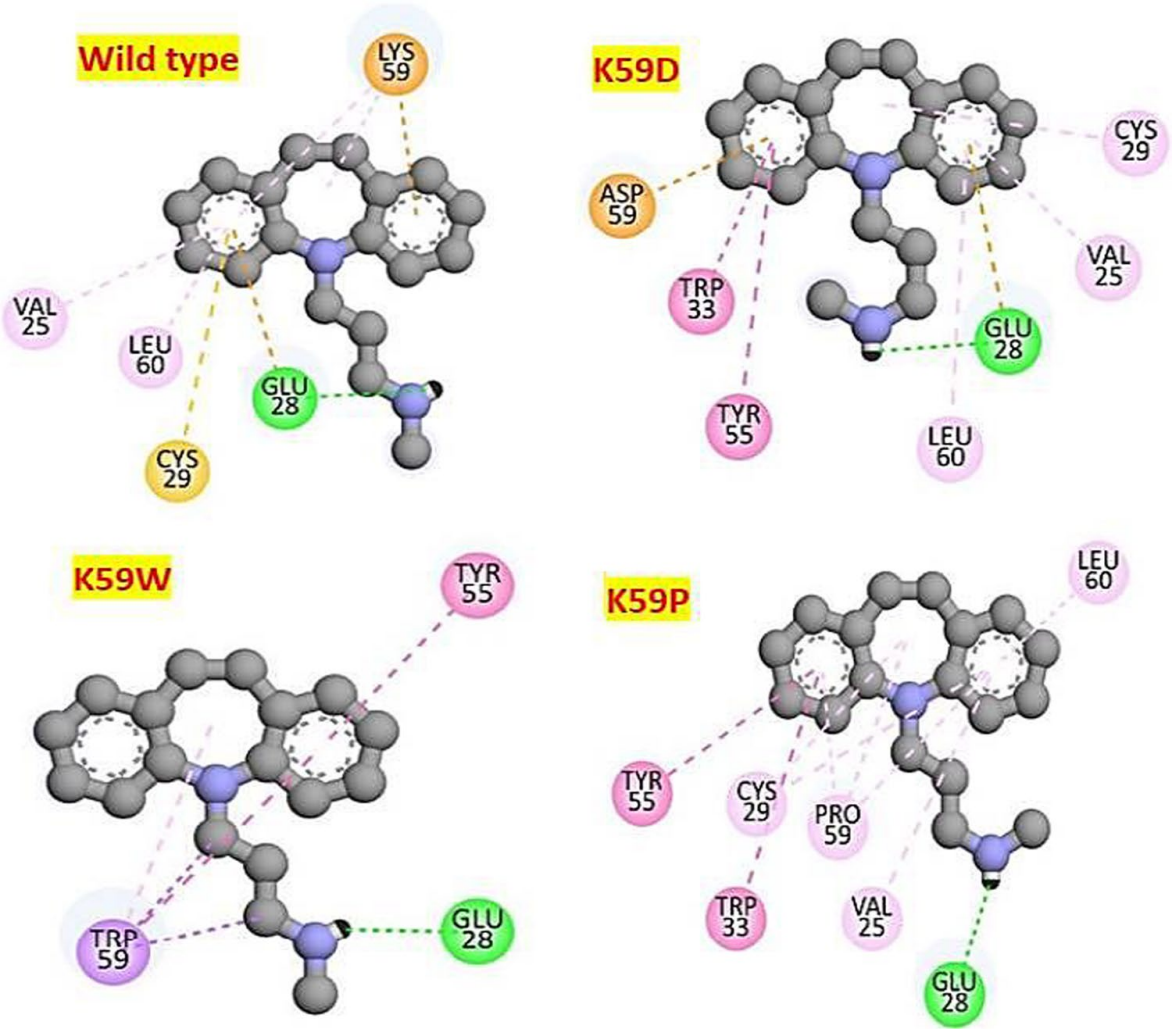
A diagram showing 2D-plots of desipramine bindings’ (having the highest ∆G values) with NNAT WT, K59D, K59W, and K59P. Coloring scheme showed green color conventional H-bonds; light green van der Waals; dark purple Pi–Pi stacked; light purple Pi-alkyl; electric blue halogen; light yellow Pi sulphur; light brown Pi-cation / Pi-anion

In this case, the quality factor for all four 3D-model (WT + all three structural modifications at K59) was overall 100, with > 92.9% residues in the most favored region of the Ramachandran plot. The significant outcome related to structural validation observed that there was no single residue existing in the generously and disallowed region of all four 3D models, which were constructed for further investigation. The investigation of the molecular interactions of all four homology models (WT, K59D, K59W, K59P) of NNAT with desipramine, preferred based on the lowest ΔG values (i.e., − 4.8, − 6.24, − 6.55, and − 5.79 kcal/mol) was done using 2D plots to explore the non-covalent interactions and hydrogen bond formation status (see Fig. 2).

### Molecular docking of NNAT–drug interactions

After investigating the CIR through the CB-dock approach, the molecular docking of all four 3D models (WT + all three structural modifications at K59) was performed with the 3D models of five therapeutic drugs used in the management of AN. The NNAT WT model exhibited less lower free energy (ΔG) values when compared with the other three structurally modified 3D models (K59D, K59P, and K59W) (see Table 6). In K59D and K59W, the highest percent change in free energy (decrease in ΔG magnitude) was computed in mirtazapine, *i.e.,* 36.67%, 23.69% while a lower range of 28%, 18.05% was found in citalopram. Comparing the K59P structural modification, the highest decrease in free energy was found in desipramine (36.46%) and the lowest decrease in ΔG of 22% was observed in citalopram (see Table 6).

**Table 6:** Molecular docking for the estimation of AN drugs binding energies with wild type and pathogenic substitution at PTM (K59) site of NNAT protein

Drugs	NNAT wild type			NNAT structural modification (K59D)				NNAT NNAT structural modification (K59P)				NNAT structural modification (K59W)			
	ΔE_MM_	ΔE _(Total internal)_	ΔG_bind_ (kcal/mol)	ΔE_MM_	ΔE _(Total internal)_	ΔG_bind_ (kcal/mol)	ΔBinding affinity (%)	ΔE_MM_	ΔE _(Total internal)_	ΔG_bind_ (kcal/mol)	%Δ Binding affinity	ΔE_MM_	ΔE _(Total Internal)_	ΔG_bind_ (kcal/mol)	%Δ Binding affinity
Amitriptyline	− 5.3	− 0.65	− 4.48	− 6.65	− 0.49	− 5.83	30.1339	− 6.59	− 0.41	− 5.77	28.7946	− 6.15	− 0.26	− 5.33	18.97
Citalopram	− 5.47	− 1.09	− 4.1	− 6.64	− 1.09	− 5.26	28.292	− 6.38	− 0.93	− 5.01	22.1951	− 6.21	− 0.94	− 4.84	18.05
Desipramine	− 5.89	− 0.67	− 4.8	− 7.34	− 0.69	− 6.24	30%	− 7.64	− 0.65	− 6.55	36.4583	− 6.88	− 0.43	− 5.79	20.63
Fluoxetine	− 6.45	− 0.92	− 4.53	− 8.02	− 1.03	− 6.1	34.6578	− 8.05	− 1.04	− 6.13	35.3201	− 7.3	− 1.13	− 5.38	18.76
Mirtazapine	− 4.39	0	− 4.39	− 6	0	-6	36.6743	− 5.82	0	− 5.82	32.574	− 5.43	0	− 5.43	23.69

### Estimation of inhibition constant (*Ki*) of NNAT protein–drug interactions

**Fig. 3:**
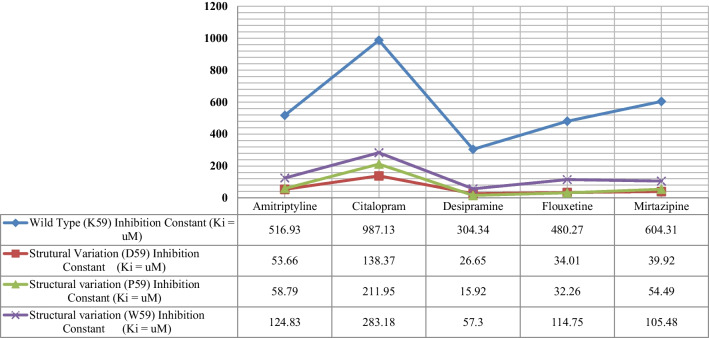
In silico computation of inhibition constant (*K*_*i*_) of AN drugs’ with NNAT WT and structural variations (K59D, P, W)

**Fig. 4:**
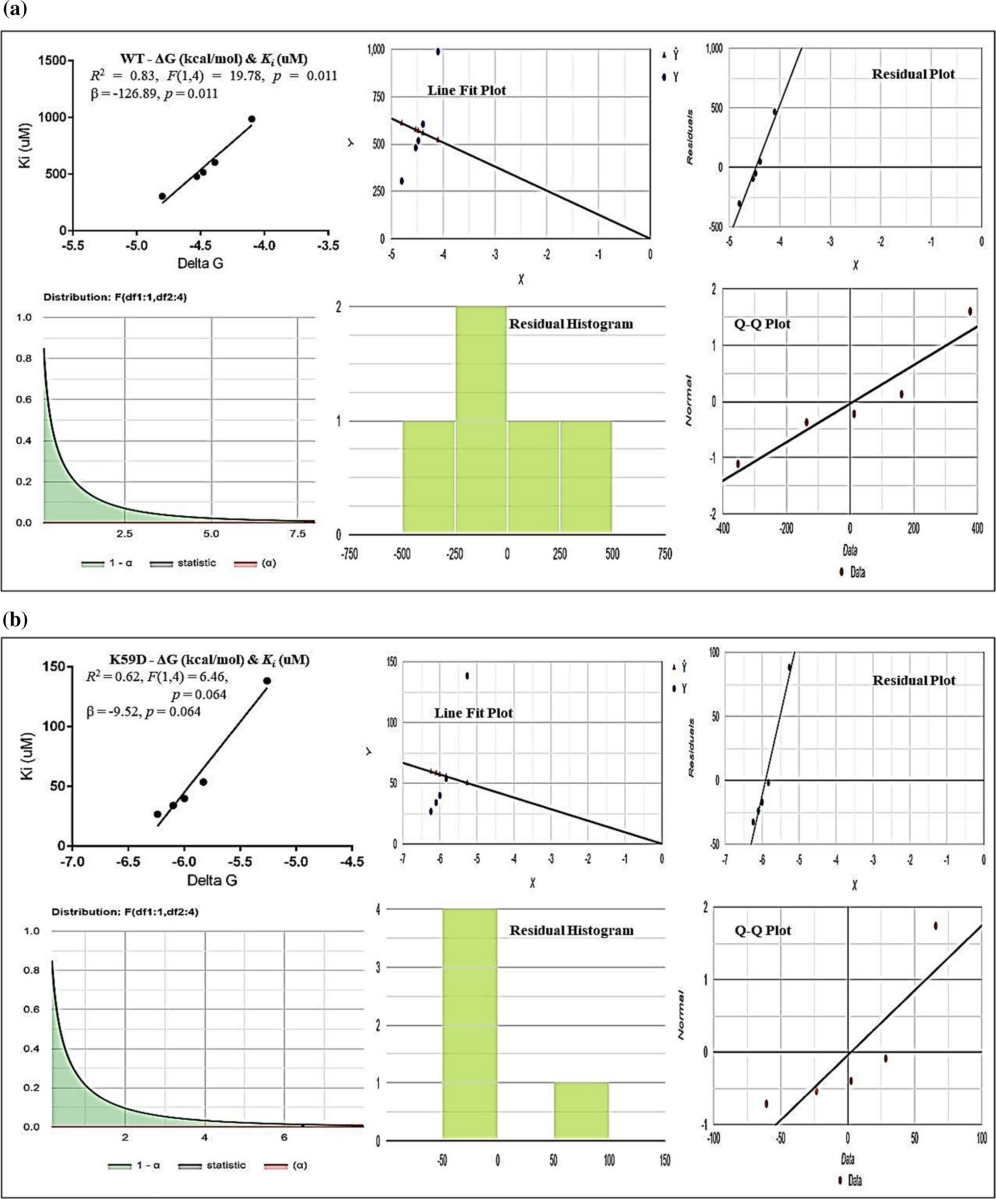

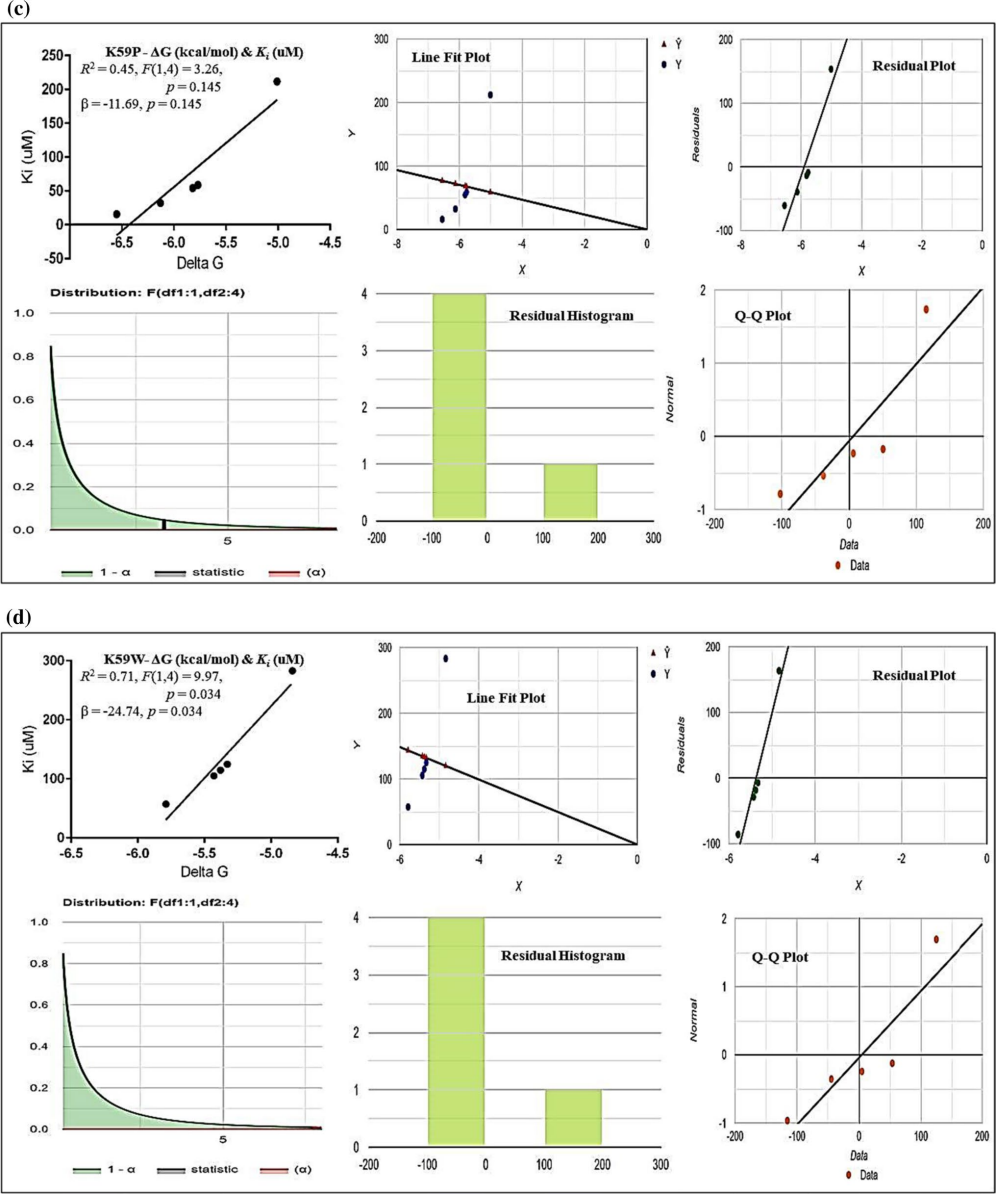
Linear regression analysis [in terms of ΔG (independent) and *Ki* (dependent)] showing the role of structural variation of different NNAT 3D models (**A** NNAT WT, **B** K59D, **C** K59P and **D** K59W) at PTM-acetylation site in drug-bindings

The values of inhibition constant (*Ki*) for all four 3D-models (WT + all three structural modifications at K59) of protein–drug interactions were computed and found to be higher in the 3D-WT model of NNAT (see Fig. 3). Very low inhibition constant (*Ki*) values were estimated in all three models of (K59D, K59P, K59P) which indicate the high binding affinities of AN drugs with the protein (see Fig. 3). The linear regression model for WT showed a strong inverse relationship (*R2* = 0.8318) which means that 83.2% of the variability of *Ki* is explained by ΔG. Shapiro (*p* value = 0.7022) which assumed that the data are normally distributed and correlation (*R*) equals − 0.912 (see Fig. 4A). For K59D, 61.8% of the variability of *Ki* is explained by ΔG (*R2* = 0.6176). Shapiro–Wilk (*p* = 0.05572) test showed that the data are normally distributed and correlation (*R*) equals − 0.7858 (see Fig. 4B). In K59P, 44.9% of the variability of *Ki* is explained by ΔG (*R2* = 0.4488), which means that the correlation (*R*) equals − 0.6699. Shapiro (*p* = 0.07499) test showed that the data are normally distributed and correlation (*R*) equals − 0.6699 (see Fig. 4C). In K59W, 71.4% of the variability of *Ki* is explained by ΔG (*R2* = 0.7137), which means that the correlation (*R*) equals − 0.8448. Shapiro (*p* = 0.1742) test showed that the data are normally distributed (see Fig. 4D).

## Discussion

AN, a biological illness of starvation that leads to malnutrition, is a neuropsychological eating disorder that often runs in families, strongly related to a genetic basis for understanding its role in disease development [12]. One of the most difficult problems in biology and medicine is figuring out and predicting the molecular causes of disease. The main significant area of research interest continues to be computational experimentation to investigate the disease-linked amino acid substitutions, especially those due to mutational consequences located at the PTM acetylation site [8, 9, 11]. PTMs processes enhance the structural as well as the functional expression of proteomes as they result in the covalent binding of functional molecules on proteins, proteolytic cleavage of regulatory domains or subunits, or degradation of complete proteins [6, 8, 11]. The study of cell biology, as well as the therapy and prevention of illness, depends on the identification and understanding of PTMs and the mutational role linked to structural modifications, as well as their impact on protein druggable targets [7, 9]. The role of PTM acetylation site loss has been established with abnormal protein expression and is the basis for several diseases [7]. Furthermore, the protein encoded by the NNAT gene is a proteolipid that may be related to regulating ion channels throughout brain development [12], and any changes in NNAT's expression (mutations) have been linked to AN [13]. In this comprehensive work, the role of structural modifications at NNAT’s PTM acetylation site was investigated in terms of their impact on residue substitution, their effect on functional stability as well as the pathogenicity of protein, and predicting the structure-variant (NNAT) role in drug-interaction regarding the management of AN.

PTM is crucial to biological processes, and its encompassing role in disease development cannot be ignored [7]. The role of non-synonymous mutations on PTM acetylation sites is required to link disease severity to changes in protein properties and functions [11]. In general, this can be a helpful aspect in estimating the site-directed gene candidates involved basis for diseases [8, 9]. Numerous SNPs in the human genome may vary from individual to individual [35]. Non-synonymous SNPs at the PTM acetylation site in a protein are typically one of the main factors contributing to such abnormal protein synthesis [8, 10, 11]. These alterations, including phosphorylation, glycosylation, ubiquitination, nitrosylation, methylation, acetylation, and proteolysis, may affect nearly every aspect of both normal cell biology and disease [9, 11]. Understanding the relationship between amino acid substitution due to genetic variants (SNPs) and the structural and functional consequences on a protein may aid in the identification of potential biomarkers for disease prognosis and diagnosis [36]. Hence, we investigate the structural modifications at the PTM acetylation site of the NNAT gene on secondary structure, torsional angles, and energetic potential to relate the significance of LYS (as wild type) at the PTM acetylation site to biological role and dynamics. Furthermore, HOPE inference also characterizes the possibilities of structural variations due to residue substitution at this PTM acetylation site conjugated with the overall stability of the protein.

In 2019, Lombardi et al. established that NNAT variants and expression changes were associated with susceptibility to eating disorders such as AN [13]. In continuation to this work, Azmi and his co-workers in 2022 reported a mutant model of the NNAT gene (C30Y) exhibiting drug binding affinity with the identification of the commonest interactions found at the PTM (acetylation) site, i.e., K59 [15]. Therefore, this work is a further extension of the previous facts, which were attempted computationally to find out the most deleterious substitution at the 59th position of the NNAT sequence (serving as the prominent acetylation PTM acetylation site) of all other amino acids in terms of their impact on the structure and function of NNAT’s role and interactions. The *MusiteDeep* analysis tool predicted that any substitution at the 59th position resulted in a loss of the PTM acetylation site on the NNAT protein, which in general negatively affects the structural confirmation of this gene [16]. Hence, this structural modification at the 59th position on the NNAT gene can also transgress the regular binding of NNAT with its respective therapeutic compounds.

Structural alterations at PTM acetylation sites due to any mutational consequences have an impact on the spatial organization of newly produced proteins, either directly or indirectly [37]. These structural changes at the PTMs site regulate biological reactions by adjusting (and sometimes turning on and off) protein-coding roles or enzyme activity [11, 37]. For regulatory aspects, these structural modifications at PTM acetylation sites on proteins have an activated (upstream) or deactivated (downstream) impact on their functional expression [37–39]. Generally, protein modifications are frequently not uniform, as a single gene might result in a confounding array of gene products due to alternative splicing and the fusion of various modifications [7, 8, 37, 39]. Thus, a relatively tiny portion of the overall quantity of the gene product may be present in a single modification state of the protein [39]. In this regard, the overall stability of a particular protein is necessary for the structural and functional role of a protein. So, we computed the impact of structural modifications at the NNAT’s PTM site for acetylation relative surface area (RSA) and accessible surface area (ASA) and overall stability, which was specifically unfavorable in ASN, ASP, CYS, GLY, HIS, ILE, PRO, SER, THR and VAL-based structural modifications at 59th position.

The PTM acetylation site in a coding gene offers surfaces with unique steric and electrostatic characteristics, and effectors’ detection of these characteristics can lead to complex interactions and subsequent events [39, 40]. Understanding the PTMs site on genes with its relevance to structural modification (mutations) is anticipated to have an impact on interactions by altering the very form of the interacting surfaces through both the non-local impacts related to the overall structure and the local difference in size from non-PTM-to PTM-amino acids [7, 8]. Moreover, loss of PTM acetylation site examples includes adding chemical groups or protein units to certain target protein residues, as well as changing a protein's charge, hydrophobicity, and structure, and adding a deleterious impact on the entire coding protein [37–40]. Therefore, to probe this impact we used a site-directed mutation (SDM) web-server tool [24] to investigate the impact of different substitutions on the overall structural environment of protein, which classified the possibilities for structural destabilization when LYS will be modified with ASN, ASP, CYS, GLY, HIS, MET, PHY, PRO, SER, THR, and VAL in terms of the computation of different structural attributes and predicted ΔΔG values.

Structural variations at the PTM acetylation site are usually linked with intrinsically disordered protein regions (IDPRs) that lack stability in secondary or tertiary structures, ultimately impacting the normal structural basis of interactions [39]. These PTM acetylation sites in genes serve as crucial regulators of different cellular processes [37]. Studies reported the pathogenic mutational impacts at PTM acetylation sites have been correlated with disease conditions, or in other words, disease-associated mutations about PTM acetylation sites were found to be enriched in mutations that change the charge, polarity, and hydrophobicity of the wild-type amino acids as well as their different physiochemical properties [7, 11, 40]. Generally, mutation-associated structural variation has an impact on the disruption of PTM acetylation sites, which might be the most prominent cause of genetic disease [7, 11, 37–40]. To validate this, the dynamic prediction tool [25] was used to compute the protein stability changes upon specific substitution at the PTM acetylation site of the NNAT gene in terms of vibrational entropy energy (ΔΔS values) between wild type and modified model and found that ARG, ASN, ASP, GLU, HIS, METH, PHE, PRO, THR, TRP, TYR, and VAL had decreased molecular flexibility.

Several examples of PTM process actively participating in various cell activities. In signaling, for instance, kinase cascades are switched on and off by the reversible addition and removal of phosphate groups [41]. Additionally, it has a significant role in cell cycle progression and cytoskeleton reorganization [42]. Acetylation plays a role in protein stability, protection of the N terminus, as well as regulation of protein–DNA interactions. Acetylation influences the control of protein–DNA interactions, N terminus protection, and protein stability via altering histones, which control transcription and cellular metabolism [43]. To probe the most deleterious residue-specific substitution at the PTM acetylation site of the NNAT gene, we used the PredictSNP consensus classifier tool [44], which revealed that three substitutions at the 59th position, ASP, PRO, and TRP instead of LYS, have a deleterious impact on the NNAT gene.

PTMs can also be present at interaction interfaces and can affect how proteins bind to one another [45]. Molecular recognition features (MoRFs) are a fascinating class of interaction sites in this context [46]. MoRFs are small, loosely organized fragments that play a crucial role in signal transduction, cell regulation, and many other processes [46]. Additionally, it was discovered that interface regions’ phosphorylation and lysine acetylation (but not ubiquitylation) sites exhibit extraordinary conservation [46]. Hence, we build a 3D model of wild type and the three models (ASP, PRO, THR) having the most pathogenic impact on substitution through computational strategies, described earlier. After a thorough 3D assessment and validation, the binding interactions of these models with therapeutic derivates of AN were investigated to compute the binding affinity and inhibition constant (*K*_*i*_). The docking output exhibited significant ΔG (kcal/mol) values with Desipramine as well as much reduced inhibition constants (*K*_*i*_) in three variant structural models, which may be interpreted as the lower the *Ki,* the higher the drug binding, compared to wild type. These larger variations in ΔG and *K*_*i*_ values serve as the most interesting attributes of structural variation at the PTM acetylation site of the NNAT gene, which in general enhances the drug-binding potential of NNAT’s variants.

### Strength and limits

In this work, the AN-associated human gene ‘NNAT’ was investigated to predict the impact of structural modifications at NNAT’s PTM-acetylation site (K59) through other amino acid substitutions instead of LYS. The PTM-acetylation site of this gene was already reported for its commonest molecular interactions/involvement when experiments were conducted with different drugs (as ligands) used to manage AN. Moreover, we computed the functional stability and increase or decrease in molecular flexibility of the NNAT protein with the wild type and structurally modified showing the possibilities of other amino acid substitutions through the computation of pathogenicity scores by various in silico machine learning algorithms.

Through consensus classifier-based estimation of the expected accuracy of modified structural models (at the 59th position), we found the three most pathogenic substitutions (i.e., PRO, THR, and ASP) with the highest pathogenic scores. We built wild-type and these three residue-specific substitution models and investigated them in terms of in silico molecular drug-binding affinities as well as inhibition (*K*_*i*_) constants with the therapeutic compounds of AN. The main strength of this work is the computation of the lowest Ki constant in structurally modified models (K59P, D, and W) as compared to the wild type, which showed that any future variation in the NNAT gene’s PTM-acetylation site in terms of the above-mentioned residues has the highest binding tendency with AN drugs. Thus, it may represent the tight binding of drug–protein interaction, thereby serving as the structural and functional basis for the propensity of AN associated with the NNAT gene. Therefore, these structural modifications at the PTM-acetylation site of the NNAT gene will be considered as a potential target to predict the impact of structural modifications on drug interaction in therapeutic intervening targets for the management of AN.

The main limitation of this work was the absence of 3D NMR/crystal structures with previously reported literature showing significant details on protein dynamics and flexibility. So far, no previous information representing the accurate molecular 3D confirmation of the NNAT gene with a prominent role of the PTM-acetylation site at the 59th position has been reported. Hence, the lack of previous data could be seen to be a gap in the study validation factor and a barrier to accurately predicting the pathophysiology of AN related to structural variants' role at the PTM-acetylation site.

### What is already known on the subject?

The protein encoded by the NNAT gene is a proteolipid that may be related to regulating ion channels throughout brain development. The role of the NNAT gene in energy metabolism has already been well established. In 2019, 2021, and 2022, Lombardi et al., Ceccarrini et al., and Azmi et al., respectively, reported the role of the NNAT gene in the pathophysiology of AN. Azmi et al., in 2022, reported the prominent drug–protein (NNAT) interactions with a mutant NNAT model (C30Y), with the most common interactions observed at the PTM-acetylation site.

### What does this study add?

In this study, the role of structural modifications on the PTM-acetylation site (59th position) of the NNAT gene was investigated. With the aid of other 19 residue-specific structural possibilities at the PTM-acetylation site, the effect of residue substitution on protein dynamics and the decrease in molecular flexibility with structural destabilization were reported. Interestingly, three residue modifications, i.e., K59D, K59P, and K59W, were found to be the most deleterious substitutions at the acetylation site of the NNAT gene. The 3D models of these structural modifications can markedly reduce the ΔG_bind_ (kcal/mol) and inhibition constants (*Ki*) compared to the wild type.

## Data Availability

The data used in the article are given with the information from where the data were taken.
